# Resource suitability drives low use of avian‐excavated tree cavities: A multi‐state occupancy dynamics approach

**DOI:** 10.1111/1365-2656.70131

**Published:** 2025-10-01

**Authors:** Diego Jhoel Zavala, Kristina Louise Cockle, Milka Raquel Gomez, Carlos Ariel Ferreyra, Eugenia Bianca Bonaparte, Facundo G. Di Sallo, Gonçalo Ferraz

**Affiliations:** ^1^ Programa de Pós‐Graduação em Ecologia, Instituto de Biociências Universidade Federal do Rio Grande do Sul Porto Alegre Brazil; ^2^ Instituto de Biología Subtropical‐CONICET‐UNaM Puerto Iguazú Misiones Argentina; ^3^ Proyecto Selva de Pino Paraná Vélez Sarsfield & San Jurjo S/N San Pedro Misiones Argentina; ^4^ Department of Forest and Conservation Sciences University of British Columbia Vancouver British Columbia Canada

**Keywords:** avian excavators, cavity dynamics, multi‐state occupancy models, nest web, resource supply, secondary cavity‐nesting birds

## Abstract

1. Avian tree‐cavity excavators are widely held to maintain diversity of forest vertebrate faunas through the facilitation of nesting resources, and yet in many systems they are absent or redundant. Why do avian excavators sometimes supply only a small proportion of cavities used by non‐excavating species?
2. Researchers hypothesized that low re‐use of excavated cavities could be driven by high rates of excavated cavity loss (low availability) or by low suitability of excavated cavities. The two hypotheses imply different cavity use dynamics.
3. The availability hypothesis predicts high rates of excavated‐cavity transition from excavators to secondary cavity‐nesters. The suitability hypothesis predicts high transition rates from excavators to empty (unused), and high reuse rates for cavities previously used by secondary cavity‐nesters.
4. From 2006 to 2021, we studied 438 excavated and non‐excavated bird nest cavities in the Atlantic Forest of Argentina, where excavators provide ~20% of cavities used by secondary cavity‐nesters. We fit our data with a multi‐state occupancy dynamics model that accounts for observation errors and estimates transition probabilities among cavity states ‘Empty’, ‘Occupied by an excavator’, ‘Occupied by a secondary cavity‐nester’ and ‘Lost’. We complemented the modelling results with a numerical simulation of cavity use dynamics.
5. As predicted by the suitability hypothesis, the estimated probability of transition was high from excavator to empty (~0.75), low from excavator to secondary cavity‐nester (~0.05) and high for reuse by secondary cavity‐nester. Transition from ‘Empty’ to use by secondary cavity‐nester was much more probable among non‐excavated (~0.2) than excavated cavities (~0.05), which—our simulation shows—is compatible with secondary cavity‐nesters using excavated cavities in proportion to their availability. Excavated cavities remained available for several years after their last use, suggesting suitability declines with age.
6. We conclude that the marginal role of excavators as cavity producers is driven primarily by low suitability of their excavated cavities for other birds. Statistical support for the suitability hypothesis relied on the quantification of uncertainty about cavity states afforded by multi‐state occupancy dynamics models. We encourage further exploration of state‐transition probabilities among tree cavities and other multi‐use resources to test ecological hypotheses and inform resource conservation policy.

## Graphical abstract

Birds that nest in tree cavities are sometimes dependent on a few cavity‐excavating bird species to make nesting sites available. Such interdependency, however, does not always materialize because excavated cavities may be unsuitable for continued use, even when available for several years after excavation.

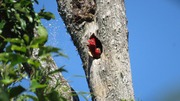

## Resumen

1. Aves excavadoras de cavidades en árboles frecuentemente proporcionan recursos de nidificación para otras aves no excavadoras, o nidificadoras secundárias. Sin embargo, en muchos sistemas ellas son ausentes o redundantes. ¿Por qué las aves no excavadoras a veces utilizan solo una pequeña proporción de las cavidades excavadas?
2. Se hipotetiza que la baja reutilización de cavidades excavadas se debe a la alta tasa de perdida (baja disponibilidad) o a la calidad inadecuada de las cavidades. Las dos hipótesis implican diferentes dinámicas de uso de cavidades.
3. Baja disponibilidad resultaría en alta tasa de transición para cavidades excavadas, de ave excavadora a nidificadora secundaria. Calidad inadecuada resultaría en alta transición de excavadora a cavidad vacía, así como alta reutilización por nidificadoras secundarias de cavidades previamente usadas por ellas.
4. Estudiamos 438 nidos de aves (2006‐2021), excavados y no‐excavados, en el Bosque Atlántico de Argentina, donde aves excavadoras proporcionan ~20% de las cavidades utilizadas por nidificadoras secundarias. Analizamos los datos con un modelo dinámico de ocupación multiestado que considera errores de observación y estima probabilidades de transición entre los estados ‘Vacía’, ‘Ocupada por excavadora’, ‘Ocupada por nidificadora secundaria’ y ’Perdida’. Complementamos los resultados con una simulación numérica de la dinámica de uso de cavidades.
5. Como predice la hipótesis de calidad, la transición estimada fue alta de ‘excavadora’ a ‘Vacía’ (~0,75), baja de ‘excavadora’ a ‘secundaria’ (~0,05) y alta para reutilización por ‘secundaria’. La transición de ‘Vacía’ a ‘secundaria’ fue más probable entre las cavidades no excavadas (~0,2) que entre las excavadas (~0,05), lo que, según nuestra simulación, es compatible con la adopción de cavidades excavadas en proporción a su disponibilidad.
6. El papel marginal de las aves excavadoras en nuestro sistema de estúdio resulta primeramente de que sus cavidades tienen baja calidad para otras aves. El soporte estadístico a la hipótesis de calidad se basa en la cuantificación de incertidumbre sobre estados de las cavidades bajo modelos dinámicos de ocupación multiestado. Recomendamos el estimado de transiciones entre estados para el estúdio de cavidades arbóreas y otros recursos de uso múltipe, tanto para testar hipótesis ecológicas como para informar estratégias de conservación.

## 1 INTRODUCTION

The processes of creation, occupancy and loss of multi‐annual nesting resources influence populations of many organisms. Yet, understanding the temporal dynamics of these resources has been a major challenge for ecologists. Various species of fish occupy and reoccupy crevices and other substrates for nesting (Mück et al., 2013); raptors and some passerines build bulky stick nests that are reused by other species (e.g. Delhey, 2018), and condors can use the same grotto for millennia (Duda et al., 2023). Some of the best studied examples of nesting resource use focus on tree cavity excavation, uptake, and re‐use as nesting sites by a wide variety of animals, including bees (Silva & Ramalho, 2014), ants (Priest et al., 2021), frogs (Mendelson et al., 2008), marsupials (Lindenmayer et al., 1991), treeshrews (Wells et al., 2006), squirrels (Bonar, 2000), weasels (Edworthy et al., 2018), bats (Kalcounis & Brigham, 1998) and almost 1900 species of birds across 72 families (van der Hoek et al., 2017). A recurring question across these taxa is as follows: what cavities are most likely to provide nesting resources, population growth and future cavity‐nester diversity? Excavator bird species (such as woodpeckers) create cavities, whereas secondary cavity‐nesters rely on existing cavities, either excavated (produced by excavators) or non‐excavated (decay‐formed), often in limited supply (Martin & Eadie, 1999). Researchers have used structural equation modelling (Blanc & Walters, 2008a), network analysis (Ruggera et al., 2016) and generalized linear models (Edworthy et al., 2012, 2018) to understand variation in rates of cavity occupancy and loss. However, the remaining challenge of quantifying probabilities of transition among resource states has prevented researchers from understanding the drivers of long‐term occupancy and predicting future resource use (e.g. Wiebe et al., 2020). Here, we use an occupancy approach to hierarchical multi‐state dynamic models, which have proven useful in understanding ecological dynamics at both the site (e.g. Martin et al., 2009) and individual levels (e.g. Lachish et al., 2011). Multi‐state occupancy dynamic models (MSODMs) account for the possibility of imperfect detection and are well suited to inferring the biological dynamics of nesting resource use (Guillera‐Arroita et al., 2014).

Avian tree‐cavity excavators are widely held to maintain diversity of cavity‐nesting faunas through the facilitation of nesting resources (Alaniz et al., 2024; Martin et al., 2004; Trzcinski et al., 2022). Cavities start with colonization of the tree by wood‐decaying fungi. Excavated cavities are formed when an avian excavator perforates the outer wall and removes decayed wood from the interior to form a cavity for nesting or roosting; non‐excavated cavities (also called ‘natural cavities’ or ‘hollows’) are enlarged, over periods of years, by the action of microorganisms, insects, fire or water (Gibbons & Lindenmayer, 2002). In some North American systems, woodpeckers produce 99% of the cavities used by secondary cavity‐nesters (Blanc & Walters, 2008b; Cadieux et al., 2023). Excavators also reuse and renovate these cavities (Wiebe et al., 2020). At global (van der Hoek et al., 2020), regional (Alaniz et al., 2024; Sandoval & Barrantes, 2009) and local scales (Blanc & Walters, 2008b; Segura, 2017), indices of abundance or of species richness of secondary cavity‐nesters often correlate with those of excavators, suggesting that excavators contribute to species diversity through the creation of tree cavities, or that excavators and secondary cavity‐nesters respond to similar habitat characteristics, like the amount of decayed wood. In temperate forests of Canada, density of excavator nests predicted the richness and density of secondary cavity‐nesting vertebrates in the following year, further supporting the notion that woodpeckers maintain forest vertebrate diversity (Trzcinski et al., 2022). Yet, in many systems globally, even where plenty of excavators are present, fewer than 1/4 of secondary cavity‐nester nests are found in excavated cavities (Altamirano et al., 2017; Bai et al., 2003; Cockle, Martin, & Wesołowski, 2011; Di Sallo & Cockle, 2022; Wesołowski, 2007).

Why do avian excavators sometimes provide only a low proportion of the nests of secondary cavity‐nesters? Two hypotheses can be considered. The cavity availability hypothesis regards all available cavities as having similar suitability. In some systems, excavated cavities are short‐lived and are found in lower supply compared to non‐excavated cavities (Cockle, Martin, & Wesołowski, 2011). Supporting this hypothesis, at least one European (Wesołowski, 2011, 2012) and two South American (Cockle et al., 2017; Paratori et al., 2023) studies report longer lifespans for non‐excavated than for excavated cavities. In contrast, the cavity suitability hypothesis posits that excavated cavities are available but infrequently used by secondary cavity‐nesters because they are not sufficiently suitable for nesting. Supporting this hypothesis, in one European study system, secondary cavity‐nesters occupied suitable non‐excavated cavities rather than available low‐quality excavated cavities (Remm et al., 2006). Previous studies have not estimated transition probabilities or accounted for imperfect detection of cavity use, potentially underestimating rates of cavity uptake and biasing results (Guillera‐Arroita, 2017). Although researchers have found some support for both hypotheses, they have struggled to weigh the evidence, which has hindered our understanding of cavity occupancy and loss, and therefore the processes that maintain populations of cavity‐nesting species.

Our multi‐state site‐occupancy analysis explores the dynamics of excavated and non‐excavated cavity use by excavating (*exc*) and secondary cavity‐nesting (*scn*) birds in the subtropical Atlantic Forest of Argentina. Our approach treats cavities as sites and nesting as site occupancy. It considers that each cavity may be found in one of four states in any given breeding season: unused (empty), used by *exc*, used by *scn*, or lost. With this setup, and accounting for the possibility of imperfect detection of cavity use, we estimate a matrix of transition probabilities between states for both types of cavities and draw on those probabilities to infer the extent to which *scn* use excavated and non‐excavated cavities. If short lifespan and low supply of excavated cavities are the main drivers of their low occupancy, we predict that excavated cavities used by *exc* should transition to *scn* use more frequently than to the empty state. In contrast, if cavity suitability is the main driver of low occupancy of excavated cavities, excavated cavities used by *exc* should transition to empty with a higher probability than to occupied by *scn*, and *scn* should hold on to their cavities (i.e. transition from *scn* to *scn*) with a higher probability in non‐excavated than in excavated cavities.

## 2 MATERIALS AND METHODS

### 2.1 Study system

Our study area spans 65 km^2^ between the towns of San Pedro (26°38′S, 54°07′ W) and Tobuna (26°27′S, 53°54′ W) in the province of Misiones, Argentina (Figure S1). The climate is subtropical with hot summers and 1200–2400 mm of rain throughout the year. The landscape includes Atlantic Forest (mostly broadleaf with the conifer *Araucaria angustifolia*), farmland and tree plantations. Selective logging occurs in some forests. Farms are ~30 ha family holdings with forest remnants and abundant isolated trees.

Among the birds nesting in the study area's tree cavities are 14 *exc* and 58 *scn* species (Supporting Information S1), along with several species of *scn* mammals and social insects (Hymenoptera). We lack estimates of cavity‐nesting species abundances for our study area, and it is inevitable that they vary in time; our field observations throughout the 16‐year study period, however, suggest that such variation did not cause substantial changes in the aggregate abundance of *scn* or *exc* species. Cavity availability in this system decreases from 4 cavities/ha in well‐conserved forest to 0.4/ha on farms (Bonaparte et al., 2020). Over <5% of the study area, 26 nest boxes were deployed in 2006 (Cockle et al., 2008) and another 64 boxes were deployed in 2007 (Cockle et al., 2010; Cockle & Bodrati, 2009). These boxes, which were not included in the present study, were used by only four cavity‐nesting species at rates that varied between 10% and 38%, consistently below the rate of tree‐cavity use (Figure S2). Boxes had mostly deteriorated beyond being usable by 2012. Despite the high diversity of excavators, in both logged and well‐conserved forest *scn* infrequently use excavated cavities (~20% of nests; Cockle, Martin, & Wesołowski, 2011) and at least some *scn* species are nest‐site limited (Cockle et al., 2010). Measurements of cavity entrance diameter, depth, height above‐ground and branch diameter overlap broadly between excavated and non‐excavated cavities, although non‐excavated cavity metrics are somewhat more variable, especially in vertical depth (Figure S3). The main differences between cavities of different origins are substrate and duration: 96% of excavated but only 24% of non‐excavated cavities were found in a dead branch or trunk; non‐excavated cavities persist longer (median: >10 years) than excavated ones (1–2 years; Cockle et al., 2017). Some large trees have more than one cavity—up to nine, in one case—but within‐tree cavity aggregation is rare. Among cavity‐bearing trees in our study system, 86% have one, 9% have two and only 5% have three or more cavities.

### 2.2 Nest cavity monitoring

We monitored nest cavities from 2006 to 2021 (Cockle et al., 2017, 2019). Each year during the bird breeding season (~September–December), a team of 2–5 observers searched for nests mainly in forests, but also in farmland. To identify cavities that potentially held an active nest, we used cues from adult bird behaviour, signs of recent excavation, nestling vocalizations and occasional information from farmers or park rangers. Nesting was visually confirmed using a 1.8‐cm diameter video camera which transmitted wirelessly to a handheld screen. In most cases, the camera was mounted on a telescoping pole (15‐m fibreglass in 2006–2015; 22‐m carbon fibre in 2016–2021). When needed, observers used a 10‐m ladder or single‐rope climbing to access cavities, inspecting their contents using a 1.6‐cm diameter video camera on a 1‐ to 3‐m flexible tube. A tree cavity was considered occupied when it contained eggs or nestlings. Inaccessible cavities (17%; higher than our poles and without a sturdy fork to support climbing) were watched or filmed (using a cell phone coupled to a telescope) for 2–6 h. We considered them occupied if a bird removed a faecal sac, brought food to the cavity or was inside for long bouts indicative of incubation or brooding. Once a cavity was deemed occupied, it entered the set of cavities that were monitored yearly (a few cavity × years were missed because of logistical constraints). Monitored cavities were checked for occupancy an average of 7 ± 7.25 times per year (mean ± SD; median 5; range: 1–80). Our final dataset contained 438 tree cavities, with 33–163 cavities visited annually (Zavala et al., 2024). Our fieldwork, which did not require ethical approval, followed the Guidelines to the Use of Wild Birds in Research (Fair et al., 2023). Field research permits were provided by the *Instituto Misionero de Biodiversidad* (01/2020, 26/21) and the *Ministerio de Ecología y RNR de la Provincia de Misiones* (024/12, 042/13, 065/14, 052/15, 050/16, 031/18, 010/19, 025/19, 01/20, 33/21 and seven unnumbered permits to Kristina Louise Cockle 2006–2011, 2017).

We consider two classes of cavity origin, excavated and non‐excavated (Cockle, Martin, & Wesołowski, 2011). Cavity origins were fixed, but cavity states could vary among years. Three cavity states were dynamic (i.e. could transition to a different state the following year): unoccupied by breeding birds (Empty), occupied by an active nest of *exc* birds (EXC), and occupied by an active nest of *scn* birds (SCN). The fourth state was static: ‘Lost’, when a cavity became completely and irreversibly inaccessible to birds, usually because the tree or cavity‐bearing limb fell (Cockle et al., 2017). Our analytical approach, described below, estimates the probabilities of cavity transition between states from 1 year to the next and how these probabilities vary according to cavity origin.

### 2.3 Data analysis

Our analysis represents cavity state dynamics using the MSODM developed by MacKenzie et al. (2009). Modelling transitions between cavity states requires using observations to estimate the state of each cavity in each year. Under our model, cavities can only be assigned one state for each year. However, if our visits did not coincide with the days the cavity was occupied, we might mistakenly assign the ‘Empty’ state to a cavity in true state EXC or SCN. Uncertainty about state estimates is informed by replicated visits to each cavity within the same year, which are treated as independent samples of the same biological reality. Because the frequency of visits varied widely among cavities, we set an arbitrary upper limit of 20 randomly selected visits for cavity × year combinations with more than 20 visits in total. We constrained our analysis to estimate the true state of each cavity for each year, starting at the year of its first visit and up to the last year of the study. This occasionally included estimating states of cavities for years in which they were not visited.

No cavity enters the dataset as ‘Lost’, but a cavity *i* that was just found may be occupied (not ‘Empty’) with probability $\psi_{i}$. If the cavity is occupied, it will be occupied by an excavating species with probability $1-\rho_{i}$, and by a *scn* with probability $\rho_{i}$:

(1) $$\;\begin{array}{c} P\left(\text{Empty}\right)=1-\psi_{i}\;\\ P\left(\mathrm{EXC}\right)=\psi_{i}\left(1-\rho_{i}\right)\\ P\left(\mathrm{SCN}\right)=\psi_{i}\rho_{i}\; \end{array}$$

As time goes by, the probability that cavity *i* transitions from state *n* to state *m* between years t and t+1, is given by $\Phi_{n,m,i,t}$, or $\Phi_{\mathrm{nm}}$, for short. In the transition matrix

(2) $${\left[\begin{array}{cccc} \Phi_{11} & \Phi_{12} & \Phi_{13} & \Phi_{14}\\ \Phi_{21} & \Phi_{22} & \Phi_{23} & \Phi_{24}\\ \Phi_{31} & \Phi_{32} & \Phi_{33} & \Phi_{34}\\ \Phi_{41} & \Phi_{42} & \Phi_{43} & \Phi_{44} \end{array}\right]}_{i,t}={\left[\begin{array}{cccc} \left(1-l_{1}\right)\left(1-b_{1}\right) & \left(1-l_{1}\right)b_{1}\left(1-s_{1}\right) & \left(1-l_{1}\right)b_{1}s_{1} & l_{1}\\ \left(1-l_{2}\right)\left(1-b_{2}\right) & \left(1-l_{2}\right)b_{2}\left(1-s_{2}\right) & \left(1-l_{2}\right)b_{2}s_{2} & l_{2}\\ \left(1-l_{3}\right)\left(1-b_{3}\right) & \left(1-l_{3}\right)b_{3}\left(1-s_{3}\right) & \left(1-l_{3}\right)b_{3}s_{3} & l_{3}\\ 0 & 0 & 0 & 1 \end{array}\right]}_{i,t},$$

rows indicate cavity states (*n* = 1,2,3,4, representing, respectively, ‘Empty’, EXC, SCN and ‘Lost’) at time t, and columns indicate the same cavity states at time t+1. A cavity that is lost in year t (Row 4) will have zero probability of transitioning to empty (Column 1), or occupied (Columns 2, 3) in year t+1; it remains at state ‘Lost’ in year t+1 with probability 1. A cavity in state ‘Empty’ (Row 1), EXC (Row 2) or SCN (Row 3) in year *t* may be lost in the year t+1 with probabilities $l_{n}$. If not lost, it will remain available (with probabilities $1-l_{n}$; Columns 1–3) and may be occupied by a bird nest of any species with probabilities $b_{n}$ (Columns 2–3), or remain unoccupied by a bird nest, with probabilities $1-b_{n}$ (Column 1). An occupied cavity can be taken by *scn* birds with probabilities $s_{n}$ (Column 3), or by *exc*, with probabilities $1-s_{n}$ (Column 2). Parameter pairs (ψ, $b_{n}$) and (ρ, $s_{n}$) allude to cavity occupancy, respectively, by any bird species or by secondary cavity‐nesting bird species. The difference between the Greek‐ and Roman‐lettered parameters is that the former identify static probabilities of initial occupancy, whereas the latter identify dynamic probabilities of transition between occupancy states.

Parameters l, b and s are estimated with random temporal variation and a fixed effect of cavity origin. The following equations describe the generalized linear mixed model for $b_{n\left[i,t\right]}$, with n=1,2,3 for cavity i and year t. Estimates of l and s follow the same mathematical structure, replacing b by the appropriate parameter in the equations. First, the linear expression for $b_{n\left[i,t\right]}$ in logit space:

(3) $$\text{logit}\left(b_{n\left[i,t\right]}\right)=\alpha_{b_{n}\left[t\right]}+\beta_{b_{n}}*O_{i},$$

has a random temporal effect for intercept, $\alpha_{b_{n}\left[t\right]}$, and a fixed effect of cavity origin, $\beta_{b_{n}}$. Covariate $O_{i}$ equals 0 when i is excavated and 1 when it is not excavated. The random effects of year follow a normal distribution:

(4) $$\alpha_{b_{n}\left[t\right]}\sim N\left(\mu_{\alpha_{b_{n}}},{\sigma^{2}}_{\alpha_{b_{n}}}\right),$$

with a uniform hyperprior for the inverse logit (‘expit’) of the mean, and a half‐Cauchy hyperprior with scale parameter 2.25^2^ for the variance, following Broms et al. (2016):

(5) $$\text{expit}\left(\mu_{\alpha_{b_{n}}}\right)\sim U\left(0,1\right),$$

(6) $${\sigma^{2}}_{\alpha_{b_{n}}}\sim \text{half}-\text{Cauchy}\left(2.25^{2}\right).$$

The prior for the linear effect of cavity origin on $\phi_{n}$ is Normal with mean 0 and variance 10:

(7) $$\beta_{b_{n}}\sim N\left(0,10\right).$$

Importantly, we also consider that the probability of detecting the true state of a cavity in a given year depends on the cavity state that year. Our model accounts for the possibility of false‐negative detection errors, which entail assigning a cavity as ‘Empty’ in a year in which it was actually (if briefly) occupied by a nest. The detection matrix:

(8) $$\Theta =\left[\begin{array}{cccc} 1 & 0 & 0 & 0\\ 1-p_{2} & p_{2} & 0 & 0\\ 1-p_{3} & 0 & p_{3} & 0\\ 0 & 0 & 0 & 1 \end{array}\right],$$

represents the probability that a cavity in a true state given by the matrix row is assigned to each state given by the matrix column in the detection process. We follow here the same ordering of states along rows and columns as in the transition matrix (2). When a visited cavity is ‘Empty’ (Row 1) or ‘Lost’ (Row 4), it is always assigned its true state. Cavities that are occupied by excavators will be assigned EXC with probability $p_{2}$ and ‘Empty’ with probability $1-p_{2}$, whereas cavities occupied by *scn* will be assigned SCN with probability $p_{3}$ and ‘Empty’ with probability $1-p_{3}$. Parameters $p_{2}$ and $p_{3}$ are estimated as fixed in time and across cavity origins, using a uniform prior on the probability space, $U\left(0,1\right)$. When one cavity was used by both excavating and *scn* species in the same breeding season, we assigned it the SCN state—this happened in four out of 1823 cavity × year combinations, always with excavated cavities that were first used by excavators. Cavities that were lost in year t after having been assigned a dynamic state in that same year, were kept in the dynamic state for year t and considered lost in t+1. We occasionally describe a probability *P* as ‘odds of *P*’, where ‘odds’ equals *P*/(1 − *P*). We also summarize some comparisons between two probabilities as ‘odds ratios’. A site‐occupancy dynamics model of cavity use could conceivably account for cavity‐nesting species traits, such as body size. As the model tracks cavities and not their occupants, however, such accounting would demand increased complexity in the modelling of transitions among cavity states or an increased number of states themselves. When modelling N discrete cavity states with one state being ‘Lost’, the number of possible transitions is ${\left(N-1\right)}^{2}$. Add to this a minimum of N−2 detection probabilities and the total number of parameters gets quickly out of hand. We thus considered only as many states as strictly necessary for testing our hypotheses.

To assess model fit, we performed a Bayesian posterior predictive check with a Freeman–Tukey discrepancy measure (Conn et al., 2018; Supporting Information S2). The procedure returns measures of discrepancy between predicted and observed data, and between predicted and simulated data. Models with a good fit to the data produce similar measures of *predicted‐observed* and *predicted‐simulated* discrepancies. We wrote our model and posterior predictive checks in the BUGS language (Lunn et al., 2000) building on code from Kéry and Royle (2021, chap. 6) and fit the model to data in a Bayesian framework using JAGS (Plummer, 2003; code in Supporting Information S3). Our inferences are based on 3000 draws from the posterior probability distribution of model parameters using an MCMC algorithm with three chains, 60,000 iterations and a burn‐in of 40,000. We considered estimates with an R‐hat lower than 1.1 to have converged and used the results to draw posterior distributions.

### 2.4 Cavity state‐change simulation

To aid interpretation of modelling results, we simulated temporal change of nest‐cavity states and compared the resulting transition matrix with that estimated from our data and model (Supporting Information S4). The simulation explored the consequences of *scn* birds occupying excavated and non‐excavated cavities in proportion to their availability. It made four simplifying assumptions: (1) the number of cavities and the number of bird pairs, of both origins and types, respectively, are constant and similar to those observed throughout the study; (2) cavities of both types are lost at the same rate as estimated from data, there being always more cavities than bird pairs to occupy them; (3) *exc* birds rarely occupy non‐excavated cavities and take precedence in occupying excavated ones; and (4) once all *exc* birds are assigned their cavities, *scn* birds select remaining cavities at random, regardless of cavity origin. This simulation was carried out independently of model fitting and has no relationship to the simulation of the posterior predictive checks.

## 3 RESULTS

Among 438 cavities monitored in 356 trees, 405 entered the dataset after the first year of the study. Eighty cavities were lost. Annually, 35%–49% of the monitored cavities were excavated by birds, the remainder being non‐excavated (Figure S2A). Each year more than half of all cavities were found to be empty. This proportion was slightly higher and more variable among excavated cavities (Figure S2B). We found 47 bird species nesting in tree cavities, including 11 excavators (*exc*), which were very rarely found in non‐excavated cavities (~3% of nests; Table S1). Nesting *scn* birds were found in excavated cavities more frequently (11% of nests) than *exc* birds in non‐excavated ones. Of the 11 *scn* species for which we found at least 10 nests, 8 were found in both types of cavities, 3 exclusively in non‐excavated cavities and none exclusively in excavated ones (Table S1). In any given year, more than a third of the nests found in excavated cavities were *exc* nests (Figure S2C).

**FIGURE 1:**
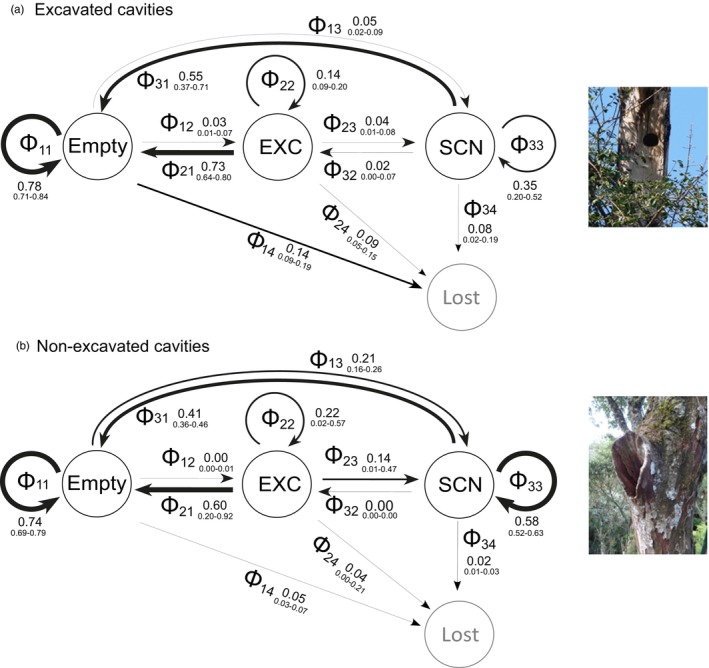
Transition diagrams for excavated (a) and non‐excavated (b) cavities showing estimated transition probabilities (Φ) among the four cavity states: ‘Empty’ (1), EXC (2), SCN (3) and ‘Lost’ (4). Indices on the Φ_nm_ parameters indicate transition from state *n* to state *m*. The values next to each Φ_nm_ symbol show the mean and 95% credible interval of the a posteriori distribution of each transition probability. Arrow widths are proportional to the magnitude of the estimated transition probability. Illustrative cavity photos by KLC.

The Bayesian posterior‐predictive‐check revealed a less‐than‐ideal, but reasonable fit of the model to data (Figure S4). Overall, *predicted*‐*observed* was higher than *predicted*‐*simulated* discrepancy, except for State 2 (EXC), where the fit was better. Because our model is simple, with straightforward assumptions, we proceeded with the analysis despite the discrepancies. In explaining multi‐state model results, we refer to the ‘outcome’ of state *x*, in a cavity that is in state *x* at time *t*, as whatever state *y* that cavity will be in at time *t* + 1. State *y* may or may not be equal to *x*, and time is measured in years. The most probable outcome of being in EXC is to become ‘Empty’; both for excavated and non‐excavated cavities ($\Phi_{21}\geq 0.6$; Figure 1). The probability that a cavity in EXC stays in EXC the following year ($\Phi_{22}$) is, on average, lower than 0.23 and statistically indistinguishable between cavity origins. The most probable outcome of being in SCN depends on origin: for excavated cavities, it is the ‘Empty’ state, with mean $\Phi_{31}\sim 0.54$; for non‐excavated cavities, it is to remain in SCN ($\Phi_{33}\sim 0.58$). The most probable outcome of being in the empty state is to remain empty ($\Phi_{11}$ >0.7 for both origins). Transitioning from ‘Empty’ to EXC ($\Phi_{12}$) is more probable for excavated (0.03) than for non‐excavated cavities (~0). Conversely, the odds of transitioning from ‘Empty’ to SCN ($\Phi_{13}$) are about five times higher for non‐excavated than for excavated cavities. Finally, the probability of transitioning to state ‘Lost’ is higher for excavated than for non‐excavated cavities in all states, but the difference is most marked in ‘Empty’, with the odds of loss being three times higher for excavated than non‐excavated cavities.

The $\Phi_{\mathrm{nm}}$ transition probabilities are functions of the parameters *l*, *b*, and *s*. Each of these, indexed by dynamic state, includes a random effect of time. These effects are all centred on zero but differ about their variances (Table 1). There is broad overlap among 95% credible intervals of the a posteriori distribution of variance estimates, but the probabilities $b_{1}$ and $b_{3}$ that, respectively, empty cavities and cavities in SCN state become occupied (by any bird), show relatively low temporal variation. The strongest evidence of temporal variation in the transition matrix is in the cavity loss probabilities $l_{1}$ and $l_{2}$ that, respectively, empty cavities and cavities in EXC state are lost from 1 year to the next.

**TABLE 1:** Estimated variance (with 95% credible intervals) of the random temporal effects on the *l*, *b* and *s* parameters which inform probabilities Φ of transition between states. Variances and their credible intervals are in logit space.

Parameter	Description of the associated probability, that cavity is …	$\hat{\sigma^{2}}$	95% C.I.
$l_{1}$	Empty in year *t*, lost in *t* + 1	1.32	0.270–4.029
$l_{2}$	Occupied by *exc* in year *t*, lost in *t* + 1	2.52	0.143–9.338
$l_{3}$	Occupied by *scn* in year *t*, lost in *t* + 1	1.25	0.003–6.203
$b_{1}$	Empty in year *t*, occupied by birds in *t* + 1	0.08	0.000–0.375
$b_{2}$	Occupied by *exc* in year *t*, occupied by birds in *t* + 1	0.59	0.001–2.926
$b_{3}$	Occupied by *scn* in year *t*, occupied by birds in *t* + 1	0.10	0.001–0.453
$s_{1}$	Empty in year *t*, occupied by *scn* in *t* + 1	1.94	0.001–11.303
$s_{2}$	Occupied by *exc* in year *t*, occupied by *scn* in *t* + 1	4.33	0.005–27.602
$s_{3}$	Occupied by *scn* in year *t*, occupied by *scn* in *t* + 1	2.28	0.003–14.032

**FIGURE 2:**
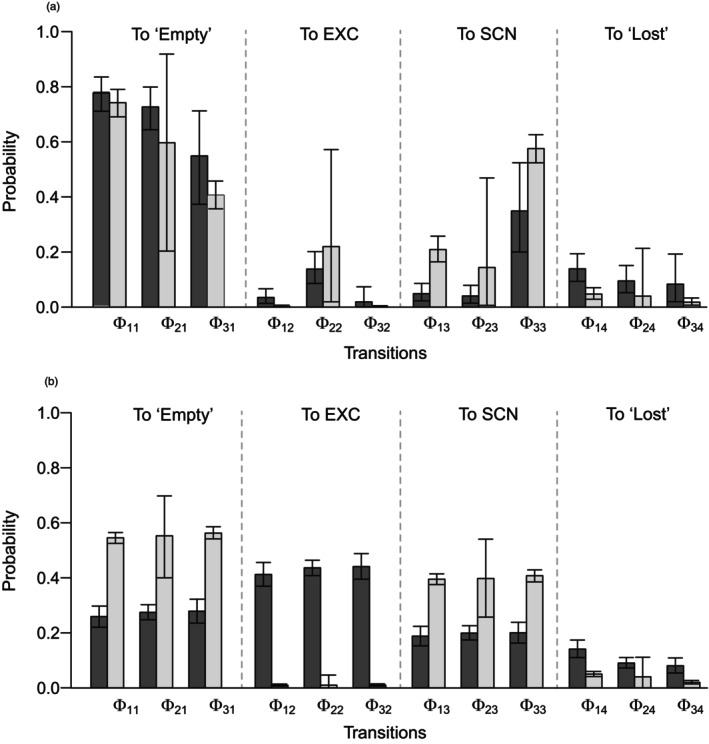
Comparison between Φ_nm_ transition probabilities estimated by the multi‐state occupancy model (a) and derived from the cavity state‐change simulations (b). Dark grey corresponds to excavated, and light grey to non‐excavated cavities. Error bars on A are the 95% credible interval of the posterior probability distribution of the parameters; on (b), they are the 0.025 and 0.975 quantiles of the distribution of transition frequencies over 3000 simulations.

**FIGURE 3:**
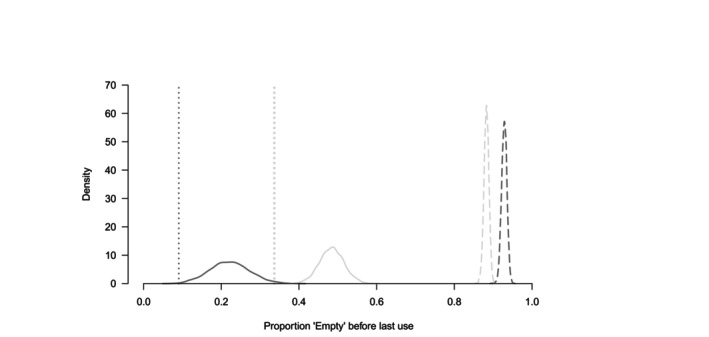
Proportion of ‘Empty’ cavity × years occurring before the final year of cavity use within each detection history. Dark grey stands for excavated, and light grey for non‐excavated cavities. Dotted lines show proportions calculated from raw data; continuous lines show the posterior probability density of the proportion from multi‐state model results; dashed lines show density of the proportion from 3000 simulations.

In both the $\Phi_{\mathrm{nm}}$ values estimated from data (Figure 2a) and those obtained from simulated nest‐cavity states (Figure 2b), the probability of transitioning from ‘Empty’ to SCN ($\Phi_{13}$) was higher for non‐excavated than for excavated cavities. The main differences between estimated and simulated transitions were that, first, probabilities of transitioning to ‘Empty’ ($\Phi_{\cdot 1}$) were higher and more variable across states when estimated from data; and second, simulated probabilities of transitioning to SCN ($\Phi_{\cdot 3}$) were indistinguishable among states within origin. In contrast and for both origins, $\Phi_{\cdot 3}$ values estimated from the data show probabilities of transitioning from SCN to SCN markedly higher than the probabilities of transitioning from EXC to SCN or from ‘Empty’ to SCN. Finally, estimated and simulated results differ markedly about the proportion of ‘Empty’ cavity × years occurring before the year of last occupancy (Figure 3). When estimated (as well as when computed directly from data) that proportion is much higher for non‐excavated than for excavated cavities. In contrast, simulations produced proportions of ‘Empty’ cavities before last use that were less variable, overall higher and relatively higher for excavated than for non‐excavated cavities.

## 4 DISCUSSION

Using a cavity‐nesting dataset from the Atlantic Forest, a multi‐state occupancy dynamics model and a state‐change simulation, our study quantified transitions between nesting‐resource states for cavity‐nesting birds and tested two hypotheses to explain differential patterns of cavity use. As predicted by the suitability hypothesis (and contrary to availability hypothesis predictions), excavated cavities used by *exc* in any given year became empty with a higher probability than they became occupied by *scn*. Also, in support of the suitability hypothesis, use by *scn* was much more persistent in non‐excavated than in excavated cavities, and excavated cavities often remained unoccupied for years after their final use, suggesting a decline in suitability with excavated cavity age. Importantly, state‐change simulation confirmed that the suitability hypothesis for low use of excavated cavities is compatible with *scn* occupying excavated and non‐excavated cavities in proportion to their availability, as observed in at least two geographically distant settings (Aitken & Martin, 2007; Cockle, Martin, & Wiebe, 2011). By quantifying transition probabilities among resource states, our study contributes to understanding why avian excavators may sometimes play a negligible role in maintaining the diversity of cavity‐nesting vertebrates.

Whereas MSODMs enable quantification of uncertainty about cavity transitions, they require the simplification of diverse multi‐species networks into a small number of states and transitions of interest. One limitation of such learning strategy is the aggregation of species according to their ability to excavate trees. Grouping species into *exc* and *scn* categories overlooks potentially interesting details of individual persistence or species turnover during a period of continued cavity use by a given category. This also ignores differences in excavation ability: large woodpeckers, such as *Campephilus* spp., can perforate harder, more intact wood (Di Sallo & Cockle, 2025) and may create longer‐lasting cavities than weaker excavators, such as Lesser Woodcreepers (*Xiphorhynchus fuscus*, Cockle et al., 2024). Moreover, species traits, such as body size, prevent certain interspecific interactions from ever occurring (e.g. a 380‐g parrot can never use a cavity excavated by a 12‐g piculet). Indeed, in a dynamic study such as ours, temporal changes in species‐specific abundance within an aggregate could produce temporal changes in dynamic parameters that would be difficult to interpret without knowledge of the underlying abundances. Nonetheless, we feel reassured in our decision to aggregate, for this study and the particular question that we are asking, because our system is diverse and because we found limited evidence of temporal variation that could be attributed to changing abundances. The minimum number of bird species necessary to accumulate 50% of the confirmed nests in our 47‐species system is seven. For comparison, the Cariboo–Chilcotin cavity nesting system of British Columbia (Martin et al., 2004) has only 19 bird species and requires nearly half as many as ours to accumulate more than half of the nests—its four most abundant species comprise 58% of the nests. Regarding temporal variation, our finding of more evidence of variability in cavity loss transitions—possibly due to exceptional weather events—than in transitions to used states, which could reflect variations in species abundance, strengthens our field impression of limited variability in the total numbers of *scn* and *exc* birds throughout the study period.

Our modelling approach can include cavity metrics as covariates of state transition (e.g. as a new term in Equation 3) but, as with nesting species, aggregation of cavities into a small set of types (e.g. excavated/non‐excavated) is parsimonious and avoids overparameterization. Such inclusion is reasonable, there being sufficient information in the data, especially when *scn* cavity suitability preferences are well documented (e.g. parrots, Renton et al., 2015; Saunders et al., 1982). We note, however, that findings from our study system have not uncovered stringent suitability preferences for the metrics that we have taken; instead, they suggest broad overlap across species within the range of cavity types and sizes compatible with body size (Bonaparte & Cockle, 2017; Cockle, Martin, & Wiebe, 2011). There is also indication of cavity suitability thresholds being context‐dependent, with some *scn* species more likely to use excavated holes in logged forest and farms than in well‐conserved forest (Bonaparte et al., 2020). As our dataset lumped a majority of cavities from well‐conserved forest with a few from disturbed areas, it would be statistically difficult to quantify context dependency. Nonetheless, the drawbacks of aggregation must be acknowledged; when possible, suitability‐based cavity selection ought to be considered with bird species‐specific studies that account for habitat context (Bonaparte et al., 2024).

By quantifying uncertainty about transitions between cavity states, we were able to compare transitions more reliably than in previous cavity‐nesting studies, which assigned cavity states without accounting for inevitable observation errors (e.g. failure to detect a nest). With the quantification of uncertainty afforded by MSODMs, researchers could more readily compare study systems along habitat gradients or among regions. They could, for example, test the hypothesis that excavators become more important cavity providers in urban and suburban habitats than in nearby, less disturbed habitats. Our approach is also applicable to testing hypotheses about the dynamics of resource transfer among organisms in other systems, including birds and bees in cliff cavities (Pacífico et al., 2020), birds in furnariid‐built nests (Delhey, 2018), epiphyte–phorophyte (Sáyago et al., 2013) and ant–plant interactions (Díaz‐Castelazo et al., 2013). Given sufficient data, MSODMs can be extended to species‐specific studies incorporating metrics of resource suitability hypothesized as relevant to the focal species. The approach can also extend to a multi‐species perspective where groups are replaced by individual species of interest, and where species could enter the model as random effects on the transition probability estimates. We encourage further exploration of state‐transition probabilities among tree cavities and other multi‐use resources, both to test ecological hypotheses about population dynamics and to inform policy regarding resource conservation.

According to our data and modelling results, use of cavities by *scn* birds was continuous, recoverable and, on the aggregate, not dependent on excavators. The odds of *scn* adopting ‘Empty’ non‐excavated cavities were 5–6.5 times higher than their odds of adopting excavated cavities that were either empty or occupied by excavators. Although we did not examine species turnover, it appears that when *scn* do occupy excavated cavities, occupancy is relatively ephemeral. The odds of re‐use by *scn* ($\Phi_{33}$) were 2.5 times higher in non‐excavated than in excavated cavities. In the circumstances of our study system, management for tree‐cavity conservation should prioritize cavities occupied by *scn* (vs. unoccupied cavities or those occupied by excavators); MSODMs can help inform such management.

Does the low probability of excavated cavity uptake by secondary cavity‐nesters conflict with the perception, already reported from our study system, that secondary cavity‐nesters use non‐excavated and excavated cavities in proportion to their availability (Cockle, Martin, & Wiebe, 2011)? Our simulation's results negate such conflict. ‘In silico’, as in nature, the frequencies of transition between cavity states reflect not just patterns of cavity selection by individuals, but also the number of birds and the availability of each cavity type. Even though (simulated) *scn* birds were blind to cavity type, simulated transitions from ‘Empty’ to SCN were still much more frequent for non‐excavated than for excavated cavities. This result does not preclude the possibility that real secondary cavity‐nesting birds prefer non‐excavated cavities, but it does show that our finding of more transitions from ‘Empty’ to SCN among non‐excavated cavities (vs. excavated cavities) is compatible with a reality in which secondary cavity‐nesters occupy excavated and non‐excavated cavities in proportion to their availability.

Trzcinski et al. (2022) proposed a fisheries‐type stock assessment model to estimate cavity availability and occupancy over time. Although their model focuses on excavated cavities, it could be extended to non‐excavated cavities and parameterized with transition probabilities from an MSODM such as ours. Such parameterization could help identify priorities for conservation of cavity‐nesting resources. For example, our results for the Atlantic Forest show that the probability of a non‐excavated cavity being occupied next year is almost three times higher for cavities used by *SCN* the previous year, versus cavities that were empty (0.58/0.21 = 2.76), suggesting that non‐excavated cavities currently occupied by secondary cavity‐nesters are the most likely to provide nesting resources, population growth and future cavity‐nester diversity. We caution, however, that a multi‐state model of cavity occupancy may tell a different story than a multi‐state model of cavity‐bearing tree use. When there is a propensity of excavators to create new cavities in the same trees, one should expect higher rates of reuse for excavated trees than for excavated cavities.

Loss of excavated cavities was more probable than loss of non‐excavated cavities ($\Phi_{\cdot 4}$, Figure 1), and yet many excavated cavities remained available long after their last use, suggesting that suitability declines with age until the cavity is eventually lost. Further strengthening the suitability hypothesis, the odds of an empty non‐excavated cavity being reoccupied (by either *exc* or *scn*) were five times higher than the odds of it being lost; for excavated cavities, on the other hand, the same odds ratio was only ~0.5, with loss more likely than reoccupation. In any given year, at least half of monitored cavities were not used, with the exception of 4 years (out of 16)—two of these being the first 2 years of observation, when most cavities were still being found. Empty cavities, excavated or not, remained empty from 1 year to the next with a probability ≥0.75. ‘Empty’ cavity × years appeared primarily after the last year of use in the raw data and in the MSODM, but primarily before the last year of cavity use in the state‐change simulation (the null expectation if cavity suitability were constant over time). Such tendency of the ‘Empty’ state to trail after the last use was particularly obvious among excavated cavities and contrasts with findings from British Columbia (Canada) where excavated cavity occupancy was 33% 3–16 years after excavation, increasing to 50% after that period (Edworthy et al., 2018). The combination of in silico cavity use dynamics and that inferred from our data strongly suggests that a considerable part of what human observers regard as available excavated cavities is not perceived as an appropriate nesting resource by secondary cavity nesting birds. Low suitability of excavated cavities makes it unlikely that excavating birds play a relevant role in regulating diversity of the cavity nesting fauna and renders the system more vulnerable to environmental disturbances that reduce the rate of decay‐based cavity formation than to the loss of particular cavity‐nesting species.

## AUTHOR CONTRIBUTIONS

Carlos Ariel Ferreyra, Milka Raquel Gomez, Kristina Louise Cockle, Facundo Di Sallo and Eugenia Bianca Bonaparte collected the data; Kristina Louise Cockle and Milka Raquel Gomez curated the data; Diego Jhoel Zavala and Gonçalo Ferraz developed statistical models and analysed the data; Diego Jhoel Zavala, Gonçalo Ferraz and Kristina Louise Cockle conceived the idea and wrote the manuscript. All authors contributed to revising the article and approved the submitted version. Our study brings together authors originally from Peru, Portugal, Canada and Argentina, with expertise in natural history, conservation biology, nest‐finding, park management and statistical ecology. Five of the seven authors are affiliated within the study region and two are park ranger technicians local to the study area. Field protocols were designed in 2006 by Kristina Louise Cockle with input from international and local collaborators. Argentine coauthors (Facundo Di Sallo, Eugenia Bianca Bonaparte, Carlos Ariel Ferreyra and Milka Raquel Gomez) contributed to the interpretation and writing of the paper. They and other Argentine collaborators developed theses, local conservation work and/or publications based on the long‐term field study. Whenever relevant, we cited literature published by scientists and naturalists from the region, including work published in Spanish.

## CONFLICT OF INTEREST STATEMENT

The authors declare no conflict of interest.

## Supporting information


**Supporting Information S1.** Bird species list.
**Table S1.** List of species present and likely to nest in tree cavities within the Misiones (Argentina) field site with occupant type as listed by Cockle et al. (2019) *Ecological Applications 29*(5), e01916. Cavity‐excavating species are listed as *exc* and secondary cavity‐nesters as *scn*. The three columns under ‘Confirmed nests’ provide information about species with nests detected in the present study. Columns show the total number of nests, as well as their partition into those in excavated and in non‐excavated cavities. Although we occasionally found bird nests inside the arboreal nests of termites and in root balls of epiphytic vegetation, we excluded these structures from the present analysis. We use a question mark (?) to indicate species that are considered likely to be *scn*, but for which there is insufficient or conflicting information about nest sites.
**Supporting Information S1.** Description of posterior predictive check procedure.
**Supporting Information S3.** Link to data and code in public repository.
**Supporting Information S4.** Description of cavity state‐change simulation.
**Figure S1.** Study area map.
**Figure S2.** Cavity type data overview.
**Figure S3.** Excavated and non‐excavated cavity metrics.
**Figure S4.** Posterior predictive check results.

## Data Availability

Data available from the Zenodo public repository: https://doi.org/10.5281/zenodo.14262784 (Zavala et al., 2024).
