# Supplementary material for: Resource suitability drives low use of avian‐excavated tree cavities: A multi‐state occupancy dynamics approach
Source: J Anim Ecol. 2025 Oct 1;94(11):2295–307. doi: 10.1111/1365-2656.70131 (PMC12586789; doi:10.1111/1365-2656.70131)
Supplement: Supplementary file 1 — Supporting Information S1. Bird species list. Table S1. List of species present and likely to nest in tree cavities within the Misiones (Argentina) field site with occupant type as listed by Cockle et al. (2019) Ecological Applications 29(5), e01916. Cavity‐excavating species are listed as exc and secondary cavity‐nesters as scn. The three columns under ‘Confirmed nests’ provide information about species with nests detected in the present study. Columns show the total number of nests, as well as their partition into those in excavated and in non‐excavated cavities. Although we occasionally found bird nests inside the arboreal nests of termites and in root balls of epiphytic vegetation, we excluded these structures from the present analysis. We use a question mark (?) to indicate species that are considered likely to be scn, but for which there is insufficient or conflicting information about nest sites. Supporting Information S1. Description of posterior predictive check procedure. Supporting Information S3. Link to data and code in public repository. Supporting Information S4. Description of cavity state‐change simulation. Figure S1. Study area map. Figure S2. Cavity type data overview. Figure S3. Excavated and non‐excavated cavity metrics. Figure S4. Posterior predictive check results. [file JANE-94-2295-s001.pdf]

SUPPLEMENTARY MATERIALS

for

Resource suitability drives low use of avian-excavated tree cavities: A multi-state occupancy dynamics approach

Table of Contents

S1. Bird species list 2

S2. Description of posterior predictive check procedure 6

S3. Link to data and code in public repository 7

S4. Description of cavity state-change simulation 8

Figure S1. Study area map.. 9

Figure S2. Cavity type data overview 10

Figure S3. Excavated and non-excavated cavity metrics 11

Figure S4. Posterior predictive check results 12

S1. Bird species list

Table S1. List of species present and likely to nest in tree cavities within the Misiones (Argentina) field site with occupant type as listed by Cockle *et al.* (2019) *Ecological Applications 29*(5), e01916. Cavity-excavating species are listed as *exc* and secondary cavity-nesters as *scn*. The three columns under “Confirmed nests” provide information about species with nests detected in the present study. Columns show the total number of nests, as well as their partition into those in excavated and in non-excavated cavities. Although we occasionally found bird nests inside the arboreal nests of termites and in root balls of epiphytic vegetation, we excluded these structures from the present analysis. We use a question mark (?) to indicate species that are considered likely to be *scn*, but for which there is insufficient or conflicting information about nest sites.

Order, family and species names	Occupant type	Confirmed nests		
		TOTAL	in excavated cavities	in non-excavated cavities
Order Cathartiformes				
Family Cathartidae				
Coragyps atratus	*scn*			
Sarcoramphus papa	*scn*			
Order Strigiformes				
Family Tytonidae				
Tyto alba	*scn*	1	0	1
Family Strigidae				
Megascops choliba	*scn*	5	2	3
Megascops atricapilla	*scn*			
Megascops sanctaecatarinae	*scn*			
Pulsatrix koeniswaldiana	*scn*			
Strix hylophila	*scn?*			
Strix virgata	*scn?*			
Glaucidium brasilianum	*scn*	21	4	17
Aegolius harrisii	*scn*	1	1	0
Order Caprimulgiformes				
Family Apodidae				
Chaetura cinereiventris	*scn*			
Chaetura meridionalis	*scn*			
Order Trogoniformes				
Family Trogonidae				
Trogon surrucura	*exc*	36	35	1
Trogon rufus	*exc*	9	8	1
Order Galbuliformes				
Family Bucconidae				
Notharchus swainsoni	*exc*			

Table S1. Continued.

Order, family and species names	Occupant type	Nests in present study		
		TOTAL	in excavated cavities	in non-excavated cavities
Order Piciformes				
Family Ramphastidae				
Ramphastos toco	*scn*			
Ramphastos dicolorus	*scn*	64	5	59
Selenidera maculirostris	*scn*	2	0	2
Pteroglossus bailloni	*scn*	9	0	9
Pteroglossus castanotis	*scn*	9	2	7
Family Picidae				
Picumnus temminckii	*exc*	18	18	0
Melanerpes flavifrons	*exc*	10	10	0
Melanerpes candidus	*exc*			
Piculus aurulentus	*exc*			
Dryobates spilogaster	*exc*	18	18	0
Campephilus robustus	*exc*	7	7	0
Dryocopus lineatus	*exc*	26	26	0
Celeus galeatus	*exc*	9	9	0
Colaptes melanochloros	*exc*	29	29	0
Colaptes campestris	*exc*	7	7	0
Order Falconiformes				
Family Falconidae				
Herpetotheres cachinnans	*scn*			
Micrastur ruficollis	*scn*	8	0	8
Micrastur semitorquatus	*scn*			
Falco rufigularis	*scn*			
Falco sparverius	*scn*	1	0	1
Order Psittaciformes				
Family Psittacidae				
Pionopsitta pileata	*scn*	9	1	8
Pionus maximiliani	*scn*	62	7	55
Amazona vinacea	*scn*	18	1	17
Pyrrhura frontalis	*scn*	91	0	91
Psittacara leucophthalmus	*scn*	69	4	65

Table S1. Continued.

Order, family and species names	Occupant type	Nests in present study		
		TOTAL	in excavated cavities	in non-excavated cavities
Order Passeriformes				
Family Grallariidae				
Grallaria varia	*scn*	3	0	3
Family Formicariidae				
Chamaeza campanisona	*scn*	46	3	43
Chamaeza ruficauda	*scn*			
Family Furnariidae				
Sittasomus griseicapillus	*scn*	16	0	16
Dendrocincla turdina	*scn*	7	1	6
Dendrocolaptes platyrostris	*scn*	24	1	23
Xiphocolaptes albicollis	*scn*	29	0	29
Xiphorhynchus fuscus	*exc*	23	20	3
Campylorhamphus falcularius	*scn*	1	0	1
Lepidocolaptes falcinellus	*scn*	3	0	3
Sclerurus scansor	*scn*			
Xenops rutilans	*scn*	1	1	0
Heliobletus contaminatus	*scn*	1	0	1
Anabacerthia lichtensteini	*scn*	6	0	6
Philydor atricapillus	*scn*			
Syndactyla rufosuperciliata	*scn*	38	5	33
Family Tityridae				
Tityra inquisitor	*scn*	6	6	0
Tityra cayana	*scn*	3	1	2
Family Tyrannidae				
Myiodynastes maculatus	*scn*	6	2	4
Myiarchus swainsoni	*scn*	5	2	3
Myiarchus ferox	*scn*			
Sirystes sibilator	*scn*			
Colonia colonus	*scn*	5	3	2
Lathrotriccus euleri	*scn*	1	0	1
Ramphotrigon megacephalum	*scn?*			
Conopias trivirgatus	*scn*			
Piprites chloris	*scn?*			

Table S1. Continued.

Order, family and species names	Occupant type	Nests in present study		
		TOTAL	in excavated cavities	in non-excavated cavities
Family Hirundinidae				
Progne chalybea	*scn*	1	1	0
Family Troglodytidae				
Troglodytes aedon	*scn*			
Family Icteridae				
Gnorimopsar chopi	*scn*	3	2	1
Family Thraupidae				
Sicalis flaveola	*scn*	1	1	0

S2. Description of posterior predictive check procedure

We assessed model fit with a Bayesian posterior predictive check using a Freeman-Tuckey (FT) discrepancy measure. Posterior predictive checks examine whether data simulated based on estimated model parameters are reasonably close to observed data, according to some aggregate descriptor of data structure. We described our data using the number of cavities observed in each of the states ‘Empty’, EXC, and SCN, in each year. For each state and year, we obtain an exact prediction of the number of observed cavities based on the estimated parameters. The FT statistic comparing this prediction with the observed data measures discrepancy between *predicted* and *observed data*. We also simulate a dataset based on the same model parameters used for the exact prediction and compute a second FT statistic that now measures discrepancy between *predicted* and *simulated data*. Models with a good fit to the data produce similar measures of *predicted-observed* and *predicted-simulated* discrepancies. FT statistics can be summed up over year and over states for different levels of generalization.

Our inference is based on 3,000 MCMC draws from the posterior probability distribution of model parameters and posterior predictive checks were performed for each MCMC iteration. Consequently, we obtained 3,000 comparisons of *predicted-observed* and *predicted-simulated* discrepancies, for each of the three dynamic states, as well as for their sum. Also, for each state and their sum, we computed a Bayesian *p*-value, given by the fraction of iterations for which *predicted-simulated* discrepancy is larger than the *predicted-observed* discrepancy.

S3. Link to data and code in public repository

https://doi.org/10.5281/zenodo.14262784

S4. Description of cavity state-change simulation

The simulation stood on four simplifying assumptions: 1) The number of cavities and the number of bird pairs that use them is constant through time, for both cavity origins and types of birds; 2) There are always more cavities than bird pairs to occupy them; 3) *exc* birds rarely occupy non-excavated cavities and take precedence in occupying excavated ones; and 4) once all *exc* birds have 'selected' their cavities, *scn* birds select cavities at random, regardless of cavity origin. We simulated cavity loss based on the l_n probabilities estimated by our model and used an arbitrary number of 200 excavated cavities to start the simulation process. All other simulation parameters were derived from the observed data. More precisely, we used the observed average ratio of 1.6 non-excavated cavities per excavated cavity to obtain the 320 non-excavated cavities in the simulation. From the average observed rates of excavated and non-excavated cavity use by *exc* and *scn* birds, we obtained the numbers of 99 excavating, and 173 non-excavating pairs of birds. We used the lowest of these rates (~1% non-excavated cavities occupied by *exc* birds in each year) to simulate the uptake of non-excavated cavities by *exc* birds (as mentioned in assumption 3, above). The simulation ran for 20 years with the following steps:

1. For each year, from 1 to 20, assign the fixed number of *exc* and *scn* pairs to cavities:

   1.1.Let non-excavated cavities be taken by *exc* pairs with a 0.01 probability.
   1.2. Count how many *exc* pairs were placed in non-excavated cavities in the previous step and attribute the remaining pairs to excavated cavities.
   1.3. Attribute all *scn* pairs to the cavities that are still available, at random, without regard to cavity origin.

2. For each year from 2 to 20 simulate cavity loss:

   2.1. Assign cavities to 'Lost' state under the probabilities l_n estimated by the model.
   2.2. Once a cavity is lost in one year, assign it to the 'Lost' state in all subsequent years.

From the matrix of cavity states through time, we then obtained a count of transitions and their respective frequencies. After repeating the process above 3,000 times, we computed the mean and quantiles of the transition frequencies, to compare with transition probabilities estimated by the model. We also computed the total proportion q of 'Empty' states that took place before the last nesting state (EXC or SCN), for both excavated and non-excavated cavities. The same proportion was computed from the observed data and from the model output. See Zenodo link in S4 for simulation code.

Figure S1. Study area map

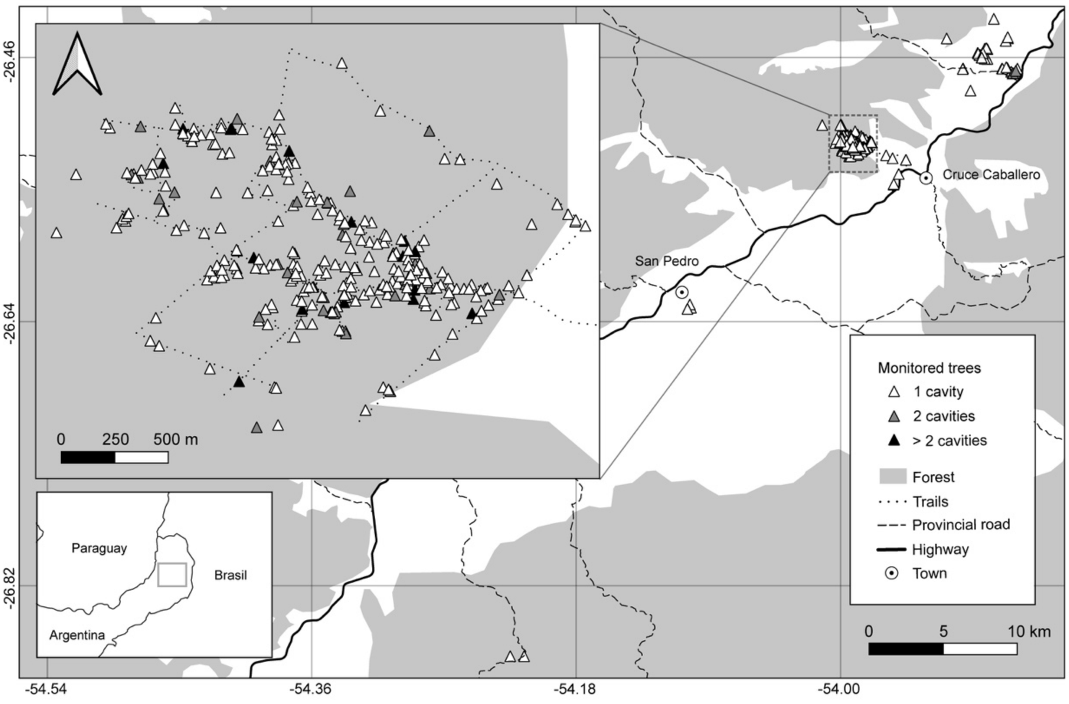

Figure S1. Study area in Misiones, Argentina, showing the spatial distribution of monitored trees with cavities. White, gray, and black triangles show trees with, respectively one, two, and more than two cavities.

Figure S2. Cavity type data overview

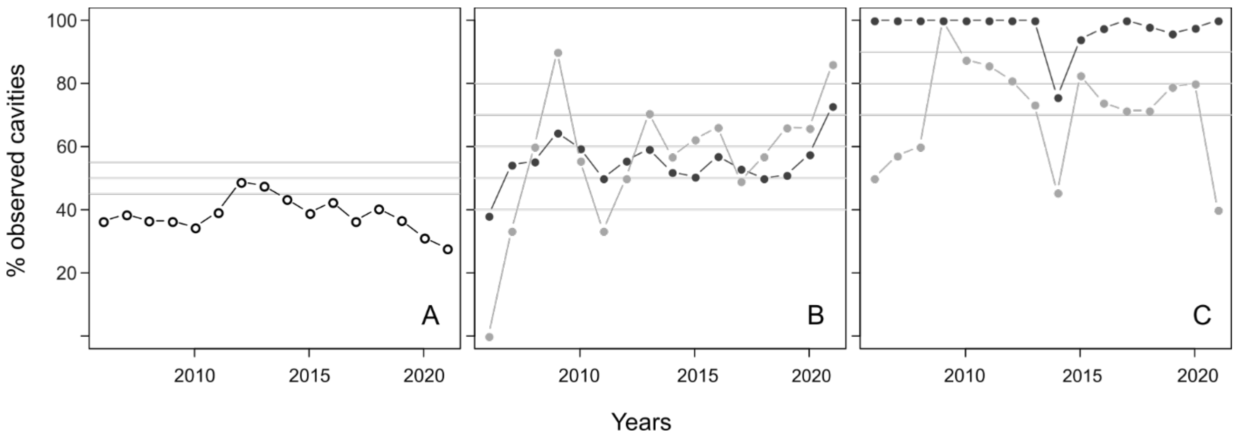

Figure S2. Overview of observed cavity types, their use, and user bird species category in each year of the sampling period: (A) proportion of all observed cavities that were excavated; (B) proportion of non-excavated (black) and excavated (gray) cavities that were found to be empty, that is, not occupied by nesting birds; and (C) observed proportion of occupied non-excavated cavities that were occupied by *scn* species (black), and the proportion of occupied excavated cavities that were occupied by *exc* species (gray). The difference between values in panel C and the 100% line on the y-axis is the proportion of occupied cavities that are occupied by the other species group. Horizontal gray lines aim to facilitate reading of y-axis values.

Figure S3. Excavated and non-excavated cavity metrics

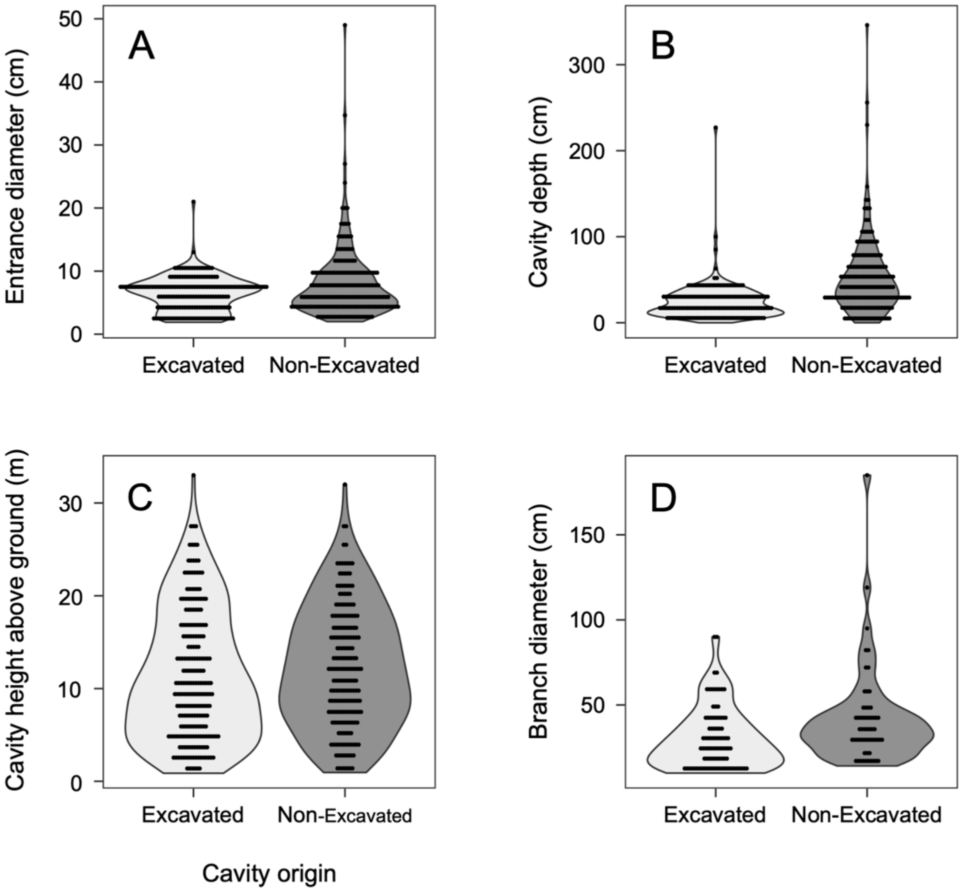

Figure S3. Distributions of entrance diameter (A), depth (B), entrance height above ground (C), and branch diameter (D) for excavated (light gray) and non-excavated (dark gray) nest cavities included in our study. Each black dot represents one measured cavity.

Figure S4. Posterior predictive check results

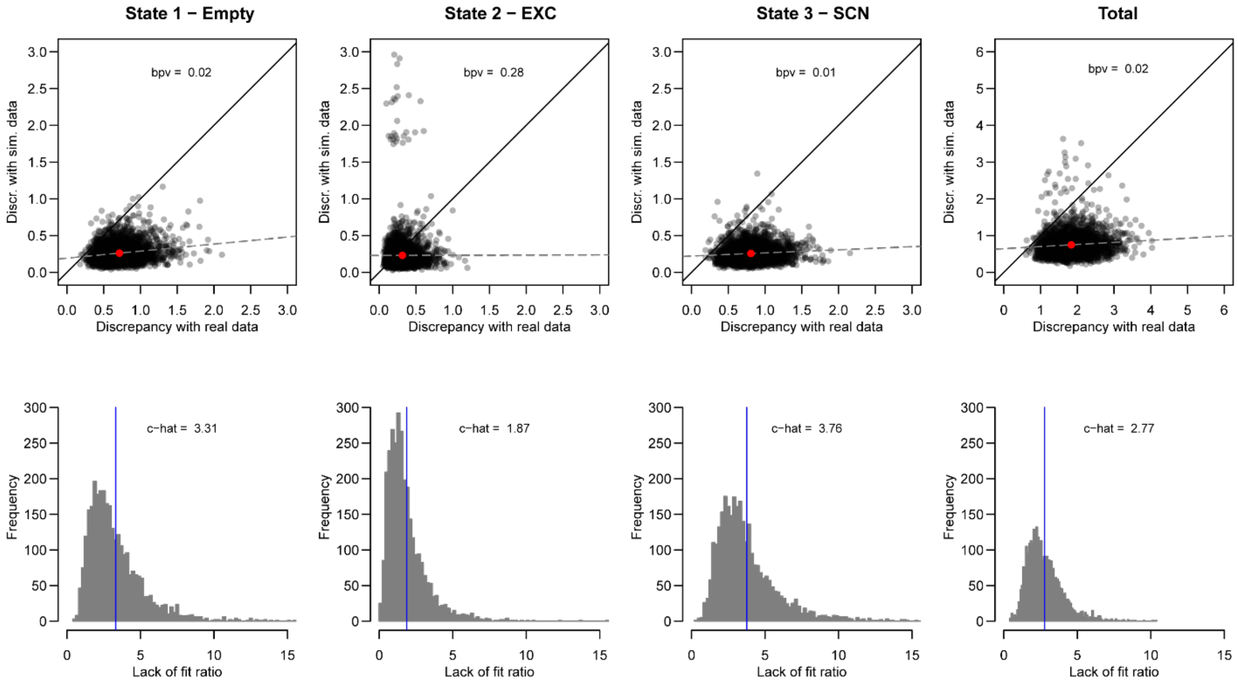

Figure S4. Bayesian posterior predictive check results. The top row shows Freeman-Tukey discrepancy metrics of *predicted-observed* discrepancy (x-axis) and *predicted*-simulated discrepancy (y-axis). Each dot is one MCMC iteration; the solid and dashed lines are, respectively, the 1:1 line, and the least squares fit to the cloud of dots. The single red dot shows the position of the mean discrepancies for both axes. The Bayesian p-value is given by 'bpv' which equals the proportion of dots that fall above the 1:1 line. The bottom row shows the frequency distribution of lack of fit ratios, where each ratio corresponds to x/y for one dot in the corresponding upper plot. The blue vertical line (c-hat) shows the frequency distribution mean. The three plot columns on the left correspond to computations done over counts of sites in sates 'Empty', EXC, and SCN. The fourth column, to the right, corresponds to the sum of Freeman-Tukey statistics for the three states on the left.
